# Evolved Eugenics and Reinforcement of “Othering”: Renewed Ethico-Legal Perspectives of Genome Editing in Reproduction

**DOI:** 10.3390/biotech12030051

**Published:** 2023-07-11

**Authors:** Pin Lean Lau

**Affiliations:** Brunel Law School, Brunel University London, Uxbridge UB8 3PH, UK; pinlean.lau@brunel.ac.uk

**Keywords:** human genome editing, germline editing, eugenics, biomedical technologies, autonomy, right to privacy, hereditary, Crispr/Cas9, reproduction, reproductive technologies

## Abstract

This article extends an exploration into renewed ethico-legal perspectives of genome editing technologies, examined from an evolved conceptualization of eugenics in contemporary human reproduction. Whilst the ethico-legal conundrums presented by genome-editing technologies in various aspects of modern medicine have thus far inspired a comprehensive trove of academic scholarship—and notwithstanding the World Health Organization’s (WHO) publication of guidelines on human genome editing in 2021—the legislative landscape for these technologies remain relatively unchanged. Accordingly, this paper presents the unresolved problematic questions that still require significant reflection. First, the paper highlights these questions, which primarily center around the tension between reproductive autonomy and the legal governance of reproductive/genome editing technologies by a democratic state. Secondly, the paper interrogates the evolved conceptualization of eugenics, exercised on the part of prospective parents as part of reproductive autonomy. By this, the paper predicates that it indirectly reinforces societal and systemic problems of discrimination and “othering”, increasing reproductive inequalities in excluded communities. Thirdly, the paper attempts to offer narratives of intersectionality as a facilitating tool in a continuing dialogue to build belonging, foster a healthy and balanced exercise of reproductive autonomy, and increase reproductive equalities.

## 1. Introduction

The Third International Summit on Human Genome Editing recently took place in March 2023 in London, England—and as international experts on human genome editing congregated at the Francis Crick Institute, what must surely be recalled in the mind was the shocking events that had unfurled at the Second International Summit on Human Genome Editing in Hong Kong in 2018. This shocking event was none other than the announcement made by Chinese biophysics scientist and researcher, Dr. He Jiankui at the second summit, that he had conducted highly secretive and allegedly successful experiments using the genome editing technology known as Crispr/Cas9, on twin embryos (effectively performing heritable human gene editing), removing the CCR5 gene in said embryos to make them resistant to HIV [1]. This immediately prompted an international outcry over what would become known as the “He Jiankui Affair”, earning He the moniker of “China’s Dr. Frankenstein”. Five years on, the He Jiankui Affair still raises antipathetic feelings, reminding us that the generational sanctity of human life continues to be vigorously safeguarded when it comes to human germline genome editing. The problems with this safeguarding, even now, are its non-legally binding nature, its reliance on the good faith of an international scientific community to uphold a consensus on moratorium [2], and most critically, its lack of mettle due to an absent international regulatory framework convention.

Whilst the He Jiankui Affair may be a disturbing true story, inspiring the streaming giant Netflix to launch a documentary titled “Make People Better” [3] and prompting the WHO to issue three reports on human genome editing, the profound consequences of the use of genome editing for human germline modification or alteration is still debated today. Indeed, in the Third International Summit on Human Genome Editing, the organizing committee of the summit issued a statement reiterating that “heritable human genome editing remains unacceptable at this time” [4]. As “governance frameworks and ethical principles for the responsible use of heritable human genome editing are not in place,” it is therefore still incumbent upon us to continue to ensure the protection of individuals from “unproven interventions in the guide of therapies” and that the international dialogues on proper governance frameworks, safety and efficacy standards, ethical approvals, and legitimate research in this field need to continue.

It would be remiss not to consider how we have arrived at this impasse. In 2020, the Nobel Prize in Chemistry was awarded to Emmanuelle Charpentier and Jennifer Doudna “for the development of a method of genome editing” [5], a revolutionary innovation known as CRISPR/Cas9 [6]. CRISPR/Cas9 is a genome editing tool that allows scientists to “edit the human genome with unprecedented precision, efficiency and flexibility” [7]. Although CRISPR had been hailed as a ground-breaking invention that could potentially transform the future of humankind by curing genetic and heritable diseases, an international scientific community at the International Summit on Human Gene Editing in Washington DC [8] in 2015 agreed that a global moratorium be imposed on human germ-line (heritable) gene editing. For the many stakeholders in the ethical, legal, social, and scientific community, germ-line gene editing is controversial for various legal and ethical reasons, amongst which, it includes the recollection of eugenic policies of various autocratic governments and a blatant disregard for human rights protections.

It is therefore unsurprising that the He Jiankui Affair was greeted with such shock and trepidation. Besides the fact that the secret experiment was highly unethical and problematic [1], it was apparent that global standards on gene editing needed to be established. After two years since the establishment of the WHO expert committee, on 12 July 2021, the WHO Expert Advisory Committee on Developing Global Standards for Governance and Oversight of Human Genome Editing (Committee) published two reports: Human Genome Editing: A Framework for Governance [9], and Human Genome Editing: Recommendations [10]. An accompanying Position Paper [11] was also published, summarizing the key points in the reports. Although other reports have been published prior to this, such as the Nuffield Council on Bioethics’ Genome Editing and Human Reproduction [12], the Committee’s reports are comprehensively unique, in that its recommendations are premised on “systems-level improvements needed to build capacity in all countries” [13]. The Committee also presented a new governance framework that builds on identifiable tools, organizations, and situations that integrate the practical difficulties of regulating human genome editing.

The Herculean task of formulating the governance framework and recommendations do not escape our admiration and is a long-awaited welcome in this field. However, it would be remiss not to question the way considerations of human rights may be factored into these recommendations. Whilst the Committee’s Recommendations incorporate hypothetical scenarios involving somatic and heritable human genome editing and proposes key ethical values and principles for use, the intrinsic human rights protections (articulated, for example, in the European Convention on Human Rights [14] or the Oviedo Convention [15]) appear to be left to the devices of institutions engaged in active governance. In LMICs (low-to-middle-income-countries) where the regulation of genome editing is not a priority or where regulation would not be in its economic interest (for example, where medical or reproductive tourism represent a lucrative commodified means of income), it would be challenging to compel compliance with the governance framework and recommendations, absent of true sanctions.

It is emphasized that the Committee’s Recommendations and framework deal with governance and that the WHO does not have the authority to regulate genome editing in individual countries. However, it also cannot be the intention that the absence of a legally positive genome editing regulation may render these Recommendations and framework unworkable in some countries due to incompatibility and hesitancy. A continuance to guarantee human rights protections in a constitutional space must be reiterated as a means of sustaining equitable governance. The key ethical values in the Recommendations can be an effective springboard to consider practical human rights questions, such as equitable access to therapies, respect for privacy and autonomy, genetic non-discrimination, and issues of disability, amongst others. Alongside adapting national systems with the Committee’s Recommendations, introducing a mechanism of “entry points of regulation” [16] relative to the role that human rights play in different constitutional systems, could be tailored by different countries to demonstrate their concerns, the “entry points” in which legally positive regulation must then be implemented.

However, these are early days yet, as the Committee continues its important work in the forthcoming months to assist the WHO in implementing the Recommendations, including building “an inclusive global dialogue on frontier technologies” [10]. As this chapter continues to unfold in the saga of human genome editing, we should continue to aspire towards achieving a truly contemporary legal application of human rights in different constitutional settings for human genome editing.

Hence, this article presents the unresolved problematic questions that still require significant reflection. First, the article highlights these questions, which primarily center around the tension between reproductive autonomy and legal governance of reproductive and genome editing technologies in reproduction by a democratic state. Secondly, the article interrogates the evolved conceptualization of eugenics, exercised on the part of prospective parents as part of reproductive autonomy. Thirdly, the article offers narratives of intersectionality as a facilitating tool in a continuing dialogue to build belonging, foster a healthy and balanced exercise of reproductive autonomy, and increase reproductive equalities.

## 2. Re-Making ‘Perfect’ Babies: Between Reproductive Autonomy and Legitimate Governance

In an earlier piece of work [7] (p. 285), this author juggled arguments that straddled Mill’s concept of liberty [14] (as applied to children) and the natural dénouement in determining the welfare of a child as being exercised by parents. Such position is that if we align with J.S. Mill’s concept of human liberty, it suggests that children, as individuals, lack the necessary capacity to exercise personal freedoms [17]. While there are criticisms of Mill’s exclusion of children from discussions about self-development and the importance of liberty and autonomy in that process, the lack of in-depth analysis in this area supports Mill’s stance on liberty [18]. Other scholarly viewpoints have accused Mill of promoting moral and legal paternalism, rejecting the idea of “adult autonomy” as a legitimate way to impose one’s choices on another, particularly on children [19]. If we accept these critiques of Mill’s stance, it would logically extend to parental decisions over their own children, going beyond the exploration of accepted parental responsibilities in natural and societal contexts [16] (p. 303).

In “normal” circumstances of child-rearing, it is challenging enough to demonstrate the development of independence in children and future generations. In matters concerning reproductive technologies and the exercise of reproductive autonomy, for instance, over the use of a preimplantation genetic diagnosis (PGD) [7], it is more complicated. If reproductive autonomy is equated to parental guidance towards a certain future plan for a child, how does one determine if this is a result of natural parenting or might be a hindrance to autonomy [16] (p. 303)? Divergent beliefs about what constitutes autonomy and who it applies to will likely lead to different responses to this question. Some parents may argue that their decisions for their children’s well-being are based on what they believe is best. In the case of being able to grant only the best human traits to children, could the desire for the “best” lead to eugenics? These questions challenge the fundamental principles of autonomy, a concept well-known in moral and legal philosophy, a prevalent topic in debates about medical treatment and individual decision-making processes. While it is easy to recognize that autonomy is needed for certain decisions, it is harder to understand its broad range in various aspects of daily life, particularly when it comes to children or future generations. Its importance is often elevated to a “supreme status”, which can shut down opposing views [7].

### 2.1. Preimplantation Genetic Diagnosis: A Gateway to Re-Making Babies?

In terms of reproductive technologies, the conceptual reproductive autonomy of parents versus legitimate state governance had already been tested earlier: when PGD emerged as a diagnostic tool that would be able to screen if embryos used in in-vitro fertilization treatments are healthy and free from genetic or other known abnormalities [16] (p. 303). PGD, which is used to screen for chromosomal abnormalities, can be more accurately targeted when specific genetic abnormalities have been identified in one or both potential parents and when couples want to prevent passing on hereditary genetic conditions to their future children. This is why PGD has been widely used in clinical settings to select healthy embryos for implantation. Single-gene disorders, such as sickle-cell anemia, cystic fibrosis, and Huntington’s disease, are examples of genetic anomalies that can be detected [20]. In these cases, PGD can be useful since it allows pre-implantation embryos to be examined in order to determine whether or not they contain the genetic material that is related to these disorders. However, more controversially, the effect of PGD is that embryos that are found to be “unhealthy” are ultimately discarded, thereby engaging questions of ethics and legality of embryo selection in this manner [21].

While PGD is becoming more popular across the globe, it is noted that it is subject to varying levels of regulation and sometimes no regulation at all in some countries. Science and technology advancements, such as CRISPR/Cas9 genome editing tools, are expected to further alter the landscape of medical and scientific treatments in the near future [7] (p. 86). It is noted that if the genome editing of embryos is a future viable option (where unhealthy embryos could potentially be fixed using technologies, such as CRISPR/Cas9), it will need to be conducted alongside PGD. This does not preclude the fact that unsuccessful attempts to repair the genetic mutations in the embryo will also still result in such embryos being discarded [22]. Hence, this could potentially impact how PGD, together with genome editing, is marketed and offered as part of fertility treatment services. This may force us to confront the difficult and highly debated ethical questions related to germline gene therapy and genetic enhancements or interventions and the possible ideation of creating designer babies [7] (p. 3).

Since the regulatory landscape for PGD is also somewhat fragmented across the globe [6], it is not surprising that there have been intense debates regarding its use. The concerns around embryo selection and the subsequent disposal of unhealthy embryos are some of the key ethical issues it raises, with prominent scientists, such as Tania Simoncelli, warning that it provides a gateway to a “new era of eugenics” [23]. Similar to the present debates surrounding human germline genome editing, PGD in its development and deployment, have been subject to the “designer babies” narrative [24].

Whilst CRISPR/Cas9 is not presently suited for commercial applications in the manner of existing fertility treatments and services and it is not likely that “designer babies” in the dystopian sense so feared by society are a possibility in the near future, the potential promise of CRISPR/Cas9 in eradicating serious genetic conditions throughout the germline [25] would hold some hope for those who suffer from such conditions. It is difficult to generate justifications for an argument that promotes gene editing for the purpose of improving a child’s chances of success in the world, versus a necessity to treat a very serious genetic condition. Any decision regarding gene editing should place the welfare and autonomy of the individual above external pressures to conform to societal success standards [26]. Additionally, gene editing does not guarantee a child’s chances of success, as success is a multifaceted concept that depends on a multitude of factors, including social and economic opportunities, personal values and aspirations, and individual abilities and abilities [27].

### 2.2. Genome Editing: Dark History, Unintended Consequences, and Reproductive Commodification

Although genome editing is presented as a panacea for many genetic ills, tinkering with our blueprint of existence raises profound moral questions that challenge our idea of what it means to be human.

This conundrum is further dominated by the specter of eugenics, when the ideology of improving humanity through selective breeding led to grave injustices and atrocities [28]. The manipulation of the human genome, even with benevolent intentions, risks resurrecting the ghosts of eugenics, as it invites the possibility of creating a genetically superior or “designer” human race [29]. This has the potential to exacerbate existing social inequalities, create a division between the genetically enhanced and the unmodified [30], and perpetuate discrimination and prejudice based on genetic makeup. The ethical implications of such a future, where access to genome editing becomes a privilege of the few, while the rest of humanity is left behind, are nothing short of dystopian [31].

Another ethical concern stems from the inherent uncertainty and potential unintended consequences of tampering with the complex web of genetic interactions. The human genome is a marvel of nature’s design [32], intricately woven together with countless genes, regulatory elements, and epigenetic modifications that influence our development, health, and identity. Editing even a single gene could have unforeseen ripple effects on the entire genome, leading to unintended consequences that may manifest in future generations [33]. The long-term effects of such alterations are largely unknown, and the potential for irreversible harm to individuals, families, and entire populations raises profound ethical dilemmas about the risks we are willing to take with the genetic heritage of humanity [34].

Furthermore, the commodification of genome editing raises troubling ethical questions about the commercialization of life itself [35]. As gene editing technologies become more accessible and market-driven, there is a risk of prioritizing profits over ethics. With the commercialization of genome editing, genetic enhancements will be available only to those who can afford them, exacerbating social inequalities and perpetuating genetic divides [36]. As technology advances, the ethical implications of turning human genes into products that can be bought and sold and the potential for exploitation and abuse raise acute concerns about the erosion of our moral compass.

In the context of reproduction, especially where the possibilities of human germline manipulation are possible, the problematic concerns surrounding genome editing are incredibly complicated and multi-factorial, harking back to Jurgen Habermas’ discourse on the future of human nature [37]. Reproduction, unfortunately, has been subject to commodification concerns throughout the course of women’s history, from reproductive tourism [38], wombs for ‘rent’ via commercial surrogacy [39], to renewed questions of making perfect babies [40] with PGD coupled with genome editing. The commodification of reproduction has emerged as a complex and contentious issue, giving rise to significant concerns with respect to its human rights implications. Central among these concerns is the potential for the exploitation of vulnerable individuals, particularly women who engage as conduit providers in reproductive services, such as egg donation or surrogacy out of financial necessity [41]. If we consider genome editing possibilities as potentially transforming the core narratives in reproductive commodification, the chasm between inequalities in reproduction will only serve to be magnified. For one, there is the disconcerting possibility that vulnerable women from socioeconomically disadvantaged backgrounds may further be exploited to take on the risks of carrying a genetically modified embryo as surrogates.

The potential commodification of human life, a similar concern (to the commodification of genome editing), raises ethical questions about the moral worth and dignity of human beings when it comes to buying and selling reproductive materials, such as gametes (eggs and sperm), embryos, or whole surrogacy arrangements [42]. When reproductive processes are reduced to commodities, genetic material and reproductive services are treated as commodities, the intrinsic value of human life and relationships become threatened [43], and widely held ethical and philosophical notions of the sanctity and inherent dignity of human beings are called into question.

In addition, the rights and welfare of children born through reproductive technologies or surrogacy also raise concerns in the context of commodification. Children conceived through these methods may face unique challenges related to their identity, origins, and relationships [44]. Questions about the genetic lineage, legal status, and nature of their relationships with the individuals involved in their conception and birth may arise, with potential implications for their human rights, including the right to know and have a relationship with their biological parents [44]. Legal frameworks governing reproductive technologies and surrogacy vary across jurisdictions and may not always adequately protect the rights and interests of these children [45]. This highlights the need for comprehensive and robust legal protections to safeguard the rights of children born through reproductive commodification.

Ultimately, the commodification of reproduction, vis-à-vis human germline genome editing, can exacerbate existing disparities and inequalities in society, as access to reproductive services may be contingent upon financial resources. This can result in a greater reproductive divide than already exists, where individuals and couples with economic means have greater access to advanced reproductive technologies, while others are left without viable options, leading to further reproductive injustice and inequality. This raises concerns about equitable access to reproductive services as a fundamental human right [46] and underscores the need to address socioeconomic disparities and ensure that all individuals have equal opportunities to exercise their reproductive choices, irrespective of their financial status [47].

### 2.3. Regulating Reproductive Autonomy in Genome Editing for Reproduction

Whilst the WHO has provided its guidance vis-à-vis the reports on standards and governance, as well as recommendations for human genome editing, leading scholars in the field have recognized that the WHO recommendation “has shifted global considerations of governing human genome editing to more pragmatic ends” [48]. Instead of recommending an outright ban on human genome editing, the WHO instead recommends that the technology be properly evaluated and “handled with care” [48]. Besides the fact that these recommendations are markedly different from the self-imposed global moratorium by the international scientific community, it also does not escape recognition that WHO recommendations do not have the force of law. Hence, considered on a global basis, it may be true to state that there is no one, unified, harmonized international law on human genome editing.

Nevertheless, it may be inferred that prior to the recommendations, there is a variety of international human rights laws [49] that either directly or indirectly have the capacity to govern genome editing [50]. For example, in the 1997 Convention for the Protection of Human Rights and Dignity of the Human Being with regard to the Application of Biology and Medicine: Convention on Human Rights and Biomedicine (the Oviedo Convention), Article 13 has usually been interpreted to mean that human genome editing is not allowed. In other soft law instruments, such as the UNESCO Universal Declaration on the Human Genome and Human Rights, Articles 1 and 10 have commonly been interpreted to emphasize that “human rights, fundamental freedoms and liberties, and human dignity, must always prevail over any research or applications that pertain to the human genome. This illustrates the respect given to key values such as personal autonomy, integrity and informed choice, especially where biology, genetics and medicine are concerned” [50].

The 2005 UNESCO Universal Declaration on Bioethics and Human Rights, in Article 2 sub-sections (d) and (f), highlights respectively, the importance of freedom of scientific research that must consider human rights and fundamental freedoms and liberties and equitable access to medical, scientific, and technological developments [50].

Verily, insofar as the domain of governance frameworks is concerned, antecedent to the WHO recommendations, some degree of apprehension and prognostication have been demonstrated regarding the path that biomedical technologies, such as genome editing tools, may traverse. Nevertheless, the actuality is that such regulations have a restricted scope, primarily when these technologies progress rapidly, and legal systems endeavor to keep pace with such developments. It becomes therefore incumbent upon us to modify the global human rights framework concurrently with the novel WHO recommendations and to work towards constructing an all-encompassing worldwide discourse on cutting-edge technologies.

In doing so, however, careful attention must be weighed between reproductive autonomy and state governance. As ethical and legal conundrums of unprecedented proportions, it is complicated, and practically impossible, to strike a balance between the sacred autonomy of parents in deciding when and how to edit their progeny’s genes and the state’s legitimate right to regulate such profound alterations of the human genome.

While parental autonomy is an essential cornerstone of personal freedom, it cannot be absolute when it comes to the manipulation of the human genome, notwithstanding that it may be for the parents’ own offspring. A legitimate interest of the state is to ensure genetic modifications do not violate fundamental ethical and human rights principles, jeopardize public health, or foster or even exacerbate existing inequalities and discrimination. It is therefore paramount that we carefully calibrate the delicate balance between parental autonomy and the state regulation of gene editing in order to preserve the sanctity of life, dignity of the human person, and the well-being of our species.

## 3. The Specter of Ghosts Past: Evolved Eugenics

The term “eugenics” is considered an almost pejorative one. Considering its association with some of the most horrific terrors that have been inflicted in human history, it is not surprising why this is the case. In any narrative that serves the improvement of human genetics, the specter of the past eugenics movement continues to haunt in several ways. First, it concerns the fear of state control over human reproduction [51]. In many ways, in contemporary democratic societies, state control already exists over reproduction. Take, for example, the United States’ Supreme Court decision in Dobbs v Jackson Women’s Health Organization [52], which effectively overturned a 1973 ruling in Roe v Wade [53] that guaranteed a constitutional right to bodily autonomy vis-à-vis the right to abortions. In many countries, women still do not have the power to realize the full extent of their sexual and reproductive rights under international law. Secondly, the fear is that genome editing could be deployed in instances “that merely deviate from a debatable genetic norm, rather than inevitably causing serious suffering” [51], and thirdly, the fear is the non-medical, non-therapeutic, genetic enhancement of characteristics, such as height, eye color, or intelligence [51]. This trio of fears is well-founded, and when viewed in context of the capabilities of genome editing, what becomes amplified is the struggle to contain the use of technologies without infringing on personal autonomy and human rights.

### 3.1. Evolved Eugenics: A Palatable Version of Its Predecessor?

Enter evolved eugenics, or as it has come to be known, “liberal eugenics” [54], where one of its strongest proponents is a prominent Professor of Ethics, Nicholas Agar. Whilst it has not been widely received, the concept of this evolved form of eugenics seeks to remove state control over reproductive choices, in favor of parental autonomy, prefaced always by the notion that the future life plan of individuals must be respected [7] (p. 54). The author of this paper states the following [7] (p. 56):

The contemporary movement of liberal eugenics, in itself, is premised on the fact that should technological advancements progress to the point of safety and availability, then parents should be at liberty to use at their disposal, the full spectrum of these technologies for the purposes of enhancement of their future offspring. The allure of liberal eugenics pivots on the centrality of this choice: the shift in autonomy from state to individual, and the freedom from state interference in its subsequent exercise by individuals. As a firm supporter of scientific and technological developments seeking to improve the quality of human life, Agar contends for the benefits that may be reaped from genetic treatments and engineering tools. Agar would be quick to argue that, should we focus on the veritable sustenance and orientation of a variety of “life plans”, the ‘new’ eugenics foothold vis-à-vis tools of genetic engineering technology, is capable of presenting adequate constrains built into the exercise of autonomy (in this regard, bearing upon the parents of the future offspring), which will not interfere into this varied projected plan of the offspring’s future, and will not be capable of directing the offspring only into the direction of one life plan.

Following the arguments of evolved eugenics, then should it not appear, that since the offensive and deplorable aspects of state-sponsored or state-sanctioned eugenics have been removed, that eugenics as we knew of, should no longer be objectionable [7] (p. 57) [19]? Be that as it may, the concept may still be extremely vertiginous to most. It also ignores the reality that, notwithstanding the purported rein of choice and freedom imbued on parents, the so-called benefits of human genome editing are not exercised by the intended beneficiary of such technology—the future offspring [7] (p. 58).

Additionally, whilst it has always been an important point that an individual’s life plan not be directed into only a limited direction, the reality is that some parental choices and actions can, and do, steer their children into specific life plans. Take the example of Harvard Girl [55], whose sole purpose of education was apparently to be accepted into the top Ivy League schools in the United States. Amy Chua’s Battle Hymn of the Tiger Mother [56] provoked controversy when it was published, revealing a list of child-rearing edicts that indirectly steered her daughters, Sophia and Lulu, into only Ivy League schools, and both achieved virtuoso pianist and violinist status. These are the realities of parental choices and actions, and even if they may not begin with the intention of limiting their children’s life plans, the consequential happenings are difficult to ignore. Hence, by which benchmark are we to determine that an individual’s life plan is suitably safeguarded? This is, in this author’s opinion, one key failing of evolved eugenics. Cloaking something deplorable with a shiny overcoat does not cease to eradicate the darkness of its history and the insurmountable limitations on life that it can bring.

Therefore, parental autonomy, choice, and actions on their own form part of parental child-rearing, which does not change the fact that future children’s life plans may already be limited. With the possibilities of a genetic supermarket being offered as enticement for human enhancement in genome editing, so too remains the limitation of a future child’s life plan; in fact, it is entirely humanly possible that the limitation of this life plan leads to further isolation and separation and perhaps objectionably, also reinforces the notion of such offspring’s “othering”—an “othering” interpreted to displace such child, guide him/her/them into a specific acceptable future life plan, all for the purpose of doing the bidding of the invisible hand of the state [7] (p. 62).

### 3.2. Contemporary Interpretations of Evolved Eugenics and “Othering”

It is the premise of this chapter to highlight a renewed angle of viewing the positive arguments towards human genome editing. Notwithstanding the allegedly more positive aspects of evolved eugenics, as used in the context here, and whilst this has somehow been equated to future offspring being better off due to the possible choices made by their parents, this author counters otherwise.

An alternative interpretation of liberal, or evolved, eugenics offered in this chapter is that the surrender of autonomy to parents is insufficient regardless, because it cannot be completely value-free of the parents’ own desires and wishes. More importantly, although bleak, as individuals existing as part of democratic societies, the existence of power relations in human interactions, vis-à-vis Foucault’s theory, is “subject to negotiation, each individual having his place in the hierarchy, no matter how flexible it would be” [57]. The purported individual control by state is otherwise wielded through “bio-power and politicization of the human body via subjugation through social and covertly political controls [58].

Foucault offers the following: “we should admit rather that power produces knowledge…; that power and knowledge directly imply one another; that there is no power relation without the correlative constitution of a field of knowledge, nor any knowledge that does not presuppose and constitute at the same time power relations; and the body” [58] (p. 25). As such, this author opines that this upholds the “politicization” of human corporeality, as postulated by Foucault, and indirectly constitutes an insidious “invisible hand” that remains under the sway of the state. Likewise, the interplay of power dynamics within the precincts of familial relations is rife, and the quest for parity between parties is unavoidably askew towards the stronger party, as evidenced by the fragmentation of authority, knowledge, and command. However, this is not to suggest that this “invisible hand” is universally deleterious. On the contrary, the author contends that some degree of state intervention is indispensable, since the discrete realm of liberal eugenics and genome editing transcends the boundaries of human existence and necessitates regulation. The thesis posited is simply that the exercise of parental autonomy is not entirely autonomous and cannot be entirely apprehended as depicted by the tenets of liberal eugenics [7] (p. 66).

In the meantime, much of the existing literature on the possible consequences of human genome editing has focused on access to technologies, inequalities, and an inevitable genetic divided [12]. Whilst this genetic divide will, no doubt, contribute to an additional layer of systematic discrimination and inequalities in society, another consequence of a genetic divide is to further magnify the problem of “othering”, a problem that, in the 21st century, we are fighting very dexterously to eradicate. It is entirely plausible that a future offspring may also experience “othering” as a consequence of being a product of genome editing. Much of existing literature has focused on the privilege of the potentially enhanced, without adequately considering the possibility that such enhancement may also create ostracization, viewing the genetically enhanced as alien to human nature. Whilst others may conject that comparisons of a genetic elite as “other” versus the systemic oppression of marginalized groups throughout history is an unfair and unbalanced rendering, our present realities of “othering” groups of individuals cannot be denied. One example of this “othering” that can be illustrated was from the recent vaccination programs for COVID-19, where anti-vaccination groups proclaimed (incorrectly) that the mRNA vaccines altered the genetic make-up of those who took said vaccines—and that this “alleged” alteration of our fundamental DNA is viewed as highly problematic and negative. Whilst this allegation proved to be untrue, what is undeniable is the way those who received the mRNA vaccines were “judged”, for willfully agreeing to the alleged tampering with the genetic make-up.

Throughout the course of history, as well, the “otherness” of being a woman, being gay, being a person with disabilities, being Roma, and being, essentially, a member of key populations [59] has been acutely felt, and in the epoch of the 21st century, “othering” continues to be a problem. Described as “a set of dynamics, processes and structures that engender marginality and persistent inequality across any of the full range of human differences based on groups identities” [60], “othering” is an unfortunate consequence of systemic discrimination and prejudice. In most cases, “othering” manifests through different ways, such as essentializing explanations [61], culturalist explanations [61] (p. 262), and racializing explanations [61] (p. 263).

Whilst “othering” can appear in many forms, including outward expressions of prejudice, it is also embedded in “institutionalization and structural features” [61] (p. 262), where “individual acts of discrimination have a cumulative and magnifying effect that may help explain many group–based inequalities” [61] (p. 263). The author posits that the genetic divide, vis-à-vis genome editing, feeds auxiliary negativity towards “othering” narratives, rendering those who cannot or do not have access to technologies, voiceless, invisible, and deviant when medicine is unable to cure them.

It is difficult to sustain the alleged benefits of evolved eugenics, even if the life plan of an individual is varied and even if parental reproductive autonomy is assumed to be the gospel truth as to what amounts to the best interest of the child [62]. At the very least, genome editing that leads to evolved eugenics in any form, will have profound implications on society, law, and policy, as Susan Stabile stipulates in the following [63]:

The contention of this Article is that an underlying attitude of “othering” pervades current discussions about what the law should and should not do to address the conditions and needs of various categories of persons. Although we do not necessarily acknowledge it, the fact that our discussions proceed from a view of the people whose situations or problems being discussed as “other” makes a difference in how we evaluate various legal and public policy initiatives. The corollary is that if, instead of proceeding from a view of others as fundamentally “not us,” we possessed an attitude of solidarity, of valuing others and seeing them as not separate or other, our views on any number of issues of public policy might be very different.

## 4. Intersectionality: Balancing the Exercise of Parental Reproductive Autonomy

In situating experiences and narratives of privilege and oppression within reproduction and, indeed, in the many facets of medicine and health, generally, this chapter recommends deeper reflections and insights into intersectionality to influence, embed, and allow for an expansion of the considerations regarding human genome editing.

The word “intersectionality” is often credited to Kimberle Crenshaw, who coined the term in 1989 [64], although it should be acknowledged that claims about the interconnectedness of race, class, gender, sexuality, and other social identities have always functioned as part of the everyday life experiences of many marginalized groups’ activities even before the term came into being. Intersectionality, added to the Oxford dictionary in 2015 is defined as “the interconnected nature of social categorizations such as race, class, and gender, regarded as creating overlapping and interdependent systems of discrimination or disadvantage”. It is a powerful analytical framework and tool for academic scholarship and a compelling driver for societal, policy, and legislative movement, change, and development. In this chapter, the author posits that the role of intersectionality goes beyond a call for equalities [65]. This chapter recommends that intersectionality, in addressing the myriad of ways genome editing technologies are made available, is critical in ways that will make us rethink how oppressive power structures are placed, how structural and systemic inequalities can permeate many aspects of just “being” and “existing”, and how we might be able to use this knowledge to reorient and center voices and experiences of marginalized communities [66].

Consistent with narratives of “othering” offered earlier, the twin diametric of privilege versus oppression plays a critical role in the under-represented picture of all communities in healthcare systems, management, quality of services, and available data. In intersectionality theory, it is critical to acknowledge that oppression does not occur in a vacuum and all types of oppression are interconnected to each other. Some of the examples of social markers, such as race, class, gender, sex, identity, socio-economic situatedness, and the like, are factors that are linked to how one experiences privilege and/or oppression. Intersectionality activists explain that in order for us to truly comprehend how oppression in society works, it means that we must always consider any type of social marker that could potentially be negatively used by oppressors to marginalize others in a community [67].

How do we do this in practice? How can we impart the reality that parental autonomy is inextricably linked to intersectionality and how it is experienced by different population groups? A starting point is education, awareness, and the openness to expand critical scientific knowledge beyond existing boundaries. This begins with the necessary acknowledgement that differences in different groups can combine and create inequalities and contribute to new movements of understanding [68]. Rascouet-Paz further states the following:

For scholars and activists, intersectionality underscores the social and political implications of categories of difference and processes of differentiation…In turn, this creates not only new avenues of inquiry but also crucial opportunities for the creation of ‘alliances, framings, and policies to address multiple inequalities.

Amidst the realm of sciences, there has been a rising clamor to integrate the concept of intersectionality as a theoretical framework in the generation of research inquiries and in the methodologies adopted. The quintessential query that comes to mind is not whether quantitative fields are capable of methodologically assimilating intersectionality, but rather if these fields are ready to broaden their definitions of epistemological methodologies so as to accommodate the intersectional inquiry in the STEM domains [69]. In essence, this necessitates a more reflected scientific inquiry, and in the context of genome editing, a conscious goal towards the equanimity of serving populations that have a necessity for such technology. Multi-stakeholder dialogues are necessitated between institutions, such as the European Medicines Agency (EMA), industry, academia, patients, and members of key population groups, in order to bolster and support the generation of data through genome editing development plans. The potential for using Health Technology Assessment (HTA) to increase financing and affordability for intersectional population groups in accessing genome editing technologies could also be explored [70].

Incorporating intersectionality into governance and regulatory frameworks for human genome editing may not provide the answers that we seek but may assist in determining how to balance parental reproductive autonomy against such governance. Though the application of genome editing for the prevention or treatment of life-threatening illnesses in unborn children seems to be an unassailable practice, it is common knowledge that distinguishing between healing and enhancement, as we travel the continuum that extends from the treatment of grave pathologies to interventions aimed at physical or cognitive refinement, is an intricate task that does not meet with unanimity among experts.

## 5. Conclusions

Genome editing technologies, and indeed, human genome editing, have rewritten the legal and ethical debates in this field [71]. Many scientists and scholars have provided compelling justification of why the highly transformative technology should be reasonably reined in to protect communities, whilst pursuing responsible and innovative research. Renewed understandings of procreative liberties and intersectionality must be suffused into the dialogue when making allowances for legal and regulatory interventions, ensuring that a healthy environment can support the thriving genome editing technologies. Simultaneously, the applications of genome editing technologies should be adequately based on proper risk and assessment, paying keen attention to international global standards of safety that have been developed, and ensuring the protection of all population groups of patients in society.

## Data Availability

No new data were created or analyzed in this study. Data sharing is not applicable to this article.

## References

[1] Sandor J. (2018). The Ethics of Genome Editing. SRHM Sex. Reprod. Health Matters.

[2] Wade N. (2018). Scientists Seek Moratorium on Edits to Human Genome That Could Be Inherited. The New York Times.

[3] Schaefer G.O. Did He Jiankui “Make People Better”? Documentary Spurs a New Look at the Case of the First Gene-Edited Babies. The Conversation. 20 December 2022. http://theconversation.com/did-he-jiankui-make-people-better-documentary-spurs-a-new-look-at-the-case-of-the-first-gene-edited-babies-196714.

[4] (2023). Statement from the Organising Committee of the Third International Summit on Human Genome Editing. https://www.theglob-alfund.org/en/key-populations/.

[5] Charpentier E., Doudna J.A. (2020). The Nobel Prize in Chemistry 2020.

[6] Hsu P.D., Lander E.S., Zhang F. (2014). Development and Applications of CRISPR-Cas9 for Genome Engineering. Cell.

[7] Lau P.L. (2019). Comparative Legal Frameworks for Pre-Implantation Embryonic Genetic Interventions.

[8] Olson S., Committee on Science, Technology, and Law, Policy and Global Affairs, and National Academies of Sciences, Engineering, and Medicine (2016). International Summit on Human Gene Editing: A Global Discussion.

[9] World Health Organization (2021). Human Genome Editing: A Framework for Governance.

[10] World Health Organization (2021). Human Genome Editing: Recommendations.

[11] World Health Organization (2021). Human Genome Editing: Position Paper.

[12] Nuffield Council on Bioethics (2018). Genome Editing and Human Reproduction: Social and Ethical Issues.

[13] World Health Organization (2021). WHO Issues New Recommendations on Human Genome Editing for the Advancement of Public Health.

[14] Conseil de l’Europe Convention for the Protection of Human Rights and Fundamental Freedoms: European Human Rights Convention (Editions du Conseil de l’Europe 1950). https://www.echr.coe.int/european-convention-on-human-rights.

[15] Conseil de l’Europe Convention for the Protection of Human Rights and Dignity of the Human Being with Regard to the Application of Biology and Medicine: Convention on Human Rights and Biomedicine (Editions du Conseil de l’Europe 1997). http://193.205.211.30/lawtech/images/lawtech/law/convenzioneoviedo.pdf.

[16] Lau P.L. (2018). The Genius & The Imbecile: Disentangling the “Legal” Framework of Autonomy in Modern Liberal Eugenics, From Non-Therapeutic Gene Enhancement Use in Gene Editing Technologies. Current Debates in International Relations and Law.

[17] Mill J.S. (1859). On Liberty.

[18] Stanley S., Mill J.S. (2017). Children’s Liberty, and the Unraveling of Autonomy. Rev. Politics.

[19] Simões M.C. (2011). Paternalism and Antipaternalism. Int. J. Moral Philos..

[20] Coggon J., Miola J. (2011). Autonomy, Liberty, and Medical Decision-Making. Camb. Law J..

[21] Basille C., Frydman R., Aly A.E., Lelorch M., Frydman N.A. (2009). Preimplantation Genetic Diagnosis: State of the Art. Eur. J. Obstet. Gynecol. Reprod. Biol..

[22] Chen H.F., Chen S.U., Ma G.C., Hsieh S.T., Tsai H.D., Yang Y.S., Chen M. (2018). Preimplantation Genetic Diagnosis and Screening: Current Status and Future Challenges. J. Formos. Med. Assoc..

[23] Simoncelli T. (2003). Pre-Implantation Genetic Diagnosis and Selection: From Disease Prevention to Customized Conception.

[24] Franklin S., Roberts C. (2006). Born and Made: An Ethnography of Preimplantation Genetic Diagnosis.

[25] Belluck P. (2017). Gene Editing for “Designer Babies”? Highly Unlikely, Scientists Say. The New York Times.

[26] Liao S., Savulescu J., Sheehan M. (2007). The Ashley Treatment: Best Interests, Convenience, and Parental Decision-Making. Hastings Cent. Rep..

[27] Sandel M. (2004). The Case against Perfection. Atl. Mon..

[28] Galton F. (1883). Inquiries into Human Faculty and Its Development.

[29] Saetz S.B., Court M.V., Henshaw M.W. (1985). Eugenics and the Third Reich.

[30] Silver L. (1997). Remaking Eden: How Genetic Engineering and Cloning Will Transform the American Family.

[31] Gaskell G., Bard I., Allansdottir A., da Cunha R.V., Eduard P., Hampel J., Hildt E., Hofmaier C., Kronberger N., Lausen S. (2017). Public Views on Gene Editing and Its Uses. Nat. Biotechnol..

[32] An Overview of the Human Genome Project (National Human Genome Research Institute (NHGRI)). https://www.genome.gov/12011238/an-overview-of-the-human-genome-project/.

[33] Ormond K.E., Mortlock D.P., Scholes D.T., Shriner D., Virani A., Young C.E. (2017). Human Germline Genome Editing. Am. J. Hum. Genet..

[34] Cribbs A.P., Perera S.M.W. (2017). Science and Bioethics of CRISPR-Cas9 Gene Editing: An Analysis Towards Separating Facts and Fiction. Yale J. Biol. Med..

[35] Maloney L. (2015). The Commodification of Human Beings. Extra Leg. Northeast. Univ. Law J..

[36] Rao R. (2006). Coercion, Commercialization, and Commodification: The Ethics of Compensation for Egg Donors in Stem Cell Research. Berkeley Technol. Law J..

[37] Habermas J. (2003). The Future of Human Nature.

[38] Voigt C., Laing J.H. (2010). Journey into Parenthood: Commodification of Reproduction as a New Tourism Niche Market. J. Travel Tour. Mark..

[39] Scott E.S. (2009). Surrogacy and the Politics of Commodification. Law Contemp. Probl..

[40] Rothschild J. (2005). The Dream of the Perfect Child.

[41] Rozée V., Sayeed Unisa S., de La Rochebrochard E. (2020). The Social Paradoxes of Commercial Surrogacy in Developing Countries: India before the New Law of 2018. BMC Women’s Health.

[42] Barroso L.R. (2012). Here, There, and Everywhere: Human Dignity in Contemporary Law and in the Transnational Discourse. Comp. Law Rev..

[43] Hurlbut J.B., Jasanoff S., Saha K. (2020). Constitutionalism at the Nexus of Life and Law. Sci. Technol. Hum. Values.

[44] Zillen K., Garland J., Slokenberga S. (2017). The Rights of Children in Biomedicine: Challenges Posed by Scientific Advances and Uncertainties.

[45] Lau P.L., Slokenberga S., Garland J. (2023). Genetic Testing or Screening at Pre-Birth Stage. Children’s Rights in Biomedicine: Protection from Scientific Risk and Uncertainty in Pursuit of the Highest Attainable Standard of Health.

[46] Galloway K. (2014). Assisted Reproductive Technologies and Human Rights. 11 Right Now. https://rightnow.org.au/opinion/assisted-reproductive-technologies-and-human-rights/.

[47] Kollodge R., Nationen V. (2017). Reproductive Health and Rights in an Age of Inequality.

[48] Cohen I.G., Sherkow J.S., Adashi E.Y. (2022). Handle with Care: The WHO Report on Human Genome Editing. Hastings Cent. Rep..

[49] Yotova R. (2020). Regulating Genome Editing under International Human Rights Law. Int. Comp. Law Q..

[50] Lau P.L. (2022). Addressing Cognitive Vulnerabilities through Genome and Epigenome Editing: Techno-Legal Adaptations for Persons with Intellectual Disabilities. Eur. J. Health Law.

[51] Coghlan N. (2023). If the EU Picks Baby Genes.

[52] Supreme Court Case: Dobbs v. Jackson Women’s Health Organization (*Center for Reproductive Rights*). https://reproductiverights.org/case/scotus-mississippi-abortion-ban/.

[53] Wade V.R. (1973). 410 U.S. Justia Law.

[54] Agar N. (1998). Liberal Eugenics. Public Aff. Q..

[55] Weihua L., Zhang Xinwu Z. (2000). Harvard Girl Liu Yiting: A Character Training Record.

[56] Chua A. (2011). Battle Hymn of the Tiger Mother.

[57] Foucault M. Naissance de La Clinique Une Archéologie Du Regard Médical. https://philpapers.org/rec/FOUNDL.

[58] Foucault M. (1977). Discipline and Punish: The Birth of The Prison.

[59] The Global Fund Key Populations. The Global Fund: Geneva, Switzerland. https://www.theglobalfund.org/en/key-populations/.

[60] Powell J.A., Menendian S. The Problem of Othering: Towards Inclusiveness and Belonging; Othering and Belonging: 2017. http://www.otheringandbelonging.org/the-problem-of-othering/.

[61] Johnson J.L., Bottorff J.L., Browne A.J., Grewal S., Hilton B.A., Clarke H. (2004). Othering and Being Othered in the Context of Health Care Services. Health Commun..

[62] Savulescu J. (2001). Procreative Beneficence: Why We Should Select the Best Children. Bioethics.

[63] Stabile S.J. (2009). Othering and the Law. St. Thomas LJ.

[64] Crenshaw K. (2017). On Intersectionality: Essential Writings.

[65] Lau P.L. (2023). Reflections on Intersectionality: Artificial Intelligence in Women’s Healthcare—Betwixt Privilege and Oppression. IV Yearbook on Socio-Economic Constitutions: Law and the Governance of Artificial Intelligence.

[66] Kelly C., Kasperavicius D., Duncan D., Etherington C., Giangregorio L., Presseau J., Sibley K.M., Straus S. (2021). ‘Doing’ or ’Using’ Intersectionality? Opportunities and Challenges in Incorporating Intersectionality into Knowledge Translation Theory and Practice. Int. J. Equity Health.

[67] Taylor B. (2019). Intersectionality 101: What Is It and Why Is It Important?.

[68] Paz R. (2020). The Indispensable Work of Understanding Intersectionality.

[69] Shattuck-Heidorn H., Boulicault M., Rushovich T., Richardson S.S. (2023). Intersectionality as Live Theory and Practice in Biomedical Sciences. The Routledge Companion to Intersectionalities.

[70] European Association of Health Law Interest Group on Supranational BioLaw (2022). Health as a Fundamental Value; Towards an Inclusive and Equitable Pharmaceutical Strategy for the European Union’.

[71] Sandor J., Slokenberga S., Minssen T., Nordberg A. (2023). Genome Editing: Learning from Its Past and Envisioning Its Future. Governing, Protecting, and Regulating the Future of Genome Editing.

